# Traumatic injury leads to ovarian cell death and reproductive disturbances in *Drosophila melanogaster*

**DOI:** 10.1242/bio.061825

**Published:** 2025-02-24

**Authors:** Cameron T. Dixon, Pamela Yang, Kimberly McCall

**Affiliations:** ^1^ Department of Biology, Boston University, Boston, MA 02215, USA; ^2^ Molecular Biology, Cell Biology and Biochemistry Program, Boston University, Boston, MA 02215, USA

**Keywords:** Traumatic injury, Reproduction, Cell death, *Drosophila*, Ovary

## Abstract

Traumatic injury (TI), or global blunt force trauma, can arise from many sources such as car crashes, sports and intimate partner violence. Effects from these injuries impact the whole organism and can lead to many different pathologies, such as inflammation, neurodegeneration, gut dysbiosis, and female reproductive detriments. *Drosophila melanogaster* has recently emerged as a powerful model to study traumatic injuries due to their high conservation of physiological effects post-trauma and the genetic toolset that they leverage. Previously, we reported female-specific reproductive deficits post mild TI in *Drosophila*. Here we investigate the effects of more severe trauma on females and found an increased retention of mature eggs and decrease in egg laying. Additionally, severe trauma led to an increase of midstage egg chamber death and formation of melanization, a known marker of immune activation. These studies provide a valuable invertebrate model to understand disturbances to female reproduction post-trauma.

## Teaser

**Summary:** Traumatic injury leads to increased ovarian cell death and disruptions in egg laying and ovulation in *Drosophila*.

## INTRODUCTION

Trauma is a leading cause of death worldwide and dwarfs the combined death tolls for contagious diseases such as HIV/AIDS, malaria, and COVID-19 (Callcut et al., 2019; Rossiter, 2022). In the United States alone, trauma is attributed to a fourth of overall causes of death for all ages (Rossiter, 2022). Traumatic injury (TI), global blunt force damage to an organism, can be caused by a wide breadth of circumstances, for example, vehicular collisions, intimate partner violence, military casualties, and high-impact sports. While death is the most severe result of TIs, individuals can develop a wide range of pathologies, including neurodegeneration, cognitive/behavioral deficits, mental illness, autoimmune disorders and metabolic disorders (Bansal et al., 2009; Shi et al., 2016). Interestingly, disruptions to homeostasis of the injured individual are not restricted to developing around the timepoint of injury and can occur later in life resulting in secondary injuries due to disruption of signaling cascades. Such cascades could include disruptions to endocrine functioning, which would have direct results on reproduction. While the majority of problems that arise post-trauma do not discriminate based on biological sex, there are a larger range of detriments specific to females. In females, TIs can lead to a breakdown of reproductive homeostasis and result in problems such as menstrual cycle dysregulation, infertility, lower libido and increased rates of miscarriages (Blaya et al., 2022; Born et al., 2006; Ripley et al., 2008). While problems with reproduction are known, the mechanisms behind these pathologies are not well understood.

Many of the established vertebrate model organisms, such as mice and zebrafish, have been utilized to understand the mechanisms behind pathologies associated with trauma (Hentig et al., 2021; Zhang et al., 2014). In recent years, the invertebrate models *Caenorhabditis elegans* and *Drosophila melanogaster* have begun to be leveraged for the trauma field and offer several benefits compared to their vertebrate counterparts (Buhlman et al., 2021; Katzenberger et al., 2015; Zanier et al., 2021). Specifically with *Drosophila*, this powerful invertebrate system has emerged as a stellar model for TI due to their highly conserved genetics and biological processes, high fecundity, short lifespan and similar physiological responses to trauma (Buhlman et al., 2021; Katzenberger et al., 2015). While many issues associated with trauma have been observed in *Drosophila*, like neurodegeneration, immune response, and metabolic disorders, it was unknown if flies shared the mammalian issues with reproduction post-trauma.

Female *Drosophila* contain two ovaries, each consisting of an average of sixteen ovarioles comprised of an assembly line of egg chamber development from the germarium to a mature egg ready to be fertilized. Each egg chamber is comprised generally of three major cell types: nurse cells (NCs), follicle cells (FCs), and the oocyte. NCs and FCs play supporting roles throughout development for the oocyte. NCs contribute vital material to the oocyte while FCs envelope and structurally maintain the developing egg. FCs also contribute to the maintenance of a healthy ovariole by removing egg chambers when either damaged or energy needs are not fulfilled (Decotto and Spradling, 2005; Lebo and McCall, 2021; Spradling et al., 1997). Egg chamber development progresses through 14 defined stages, which can be grouped as early, mid, and late stages (King, 1970; Markow, 2015; Sun and Spradling, 2013). Early and midstages have checkpoints for proper development, nutritional needs, and other environmental factors while late stages comprise programmed cell death of NCs and finalization of the oocyte (Lebo and McCall, 2021; McCall, 2004). Finally, once the developing egg chamber has successfully passed through 14 stages of oogenesis, the mature egg is then fertilized and laid (Sun and Spradling, 2013).

Previously, we found that female *Drosophila* that underwent mild trauma developed retention of mature eggs (Dixon and McCall, 2023), which is a sign of defects in ovulation (Sun and Spradling, 2013). Ovulation in *Drosophila* is controlled by secretory cells in the reproductive tract, and loss of these cells leads to decreased egg laying (Sun and Spradling, 2013), which is related to the retention of mature eggs (Grmai et al., 2024). Decreased ovulation can also be triggered by stress through high levels of ecdysone or decreased ecdysis triggering hormone, which affects neuronal inputs to egg laying and retention (Meiselman et al., 2018). To further characterize the breadth of *Drosophila* female reproductive dysfunction post-trauma, we increased the severity of trauma to determine whether there are additional consequences. During this study, we found an increase in severity of known reproductive issues in *Drosophila* (retention of mature eggs and decrease in egg laying) and identified two additional phenotypes affecting females post-trauma. These phenotypes included an increase in midstage egg chamber death and development of melanization, a hallmark of immune activation. This work demonstrates that invertebrates share reproductive defects related to trauma and establishes *Drosophila* as a valuable invertebrate model to investigate female reproductive problems post-trauma.

## RESULTS

### Females inflicted with severe trauma have increased reproductive defects

**Fig. 1.**
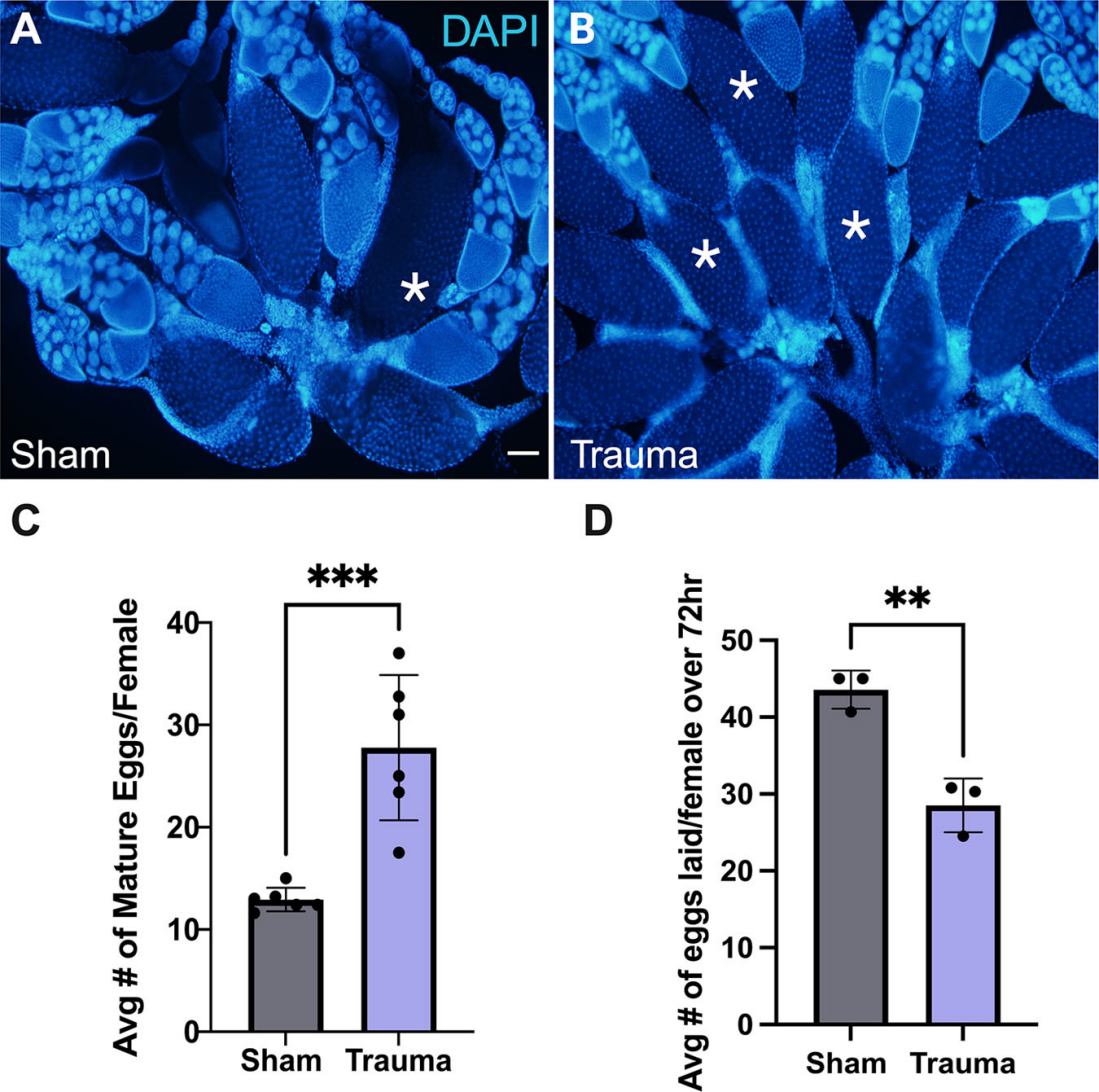
**Female *Drosophila* that undergo severe trauma show increased egg retention and decreased fecundity.** (A) Dissected ovaries from sham females [5 days post trauma (dpt)] stained with DAPI (blue) to visualize nuclei. (B) Dissected ovaries from trauma females (5 dpt). Examples of mature egg chambers are marked with asterisks (A,B). (C) Quantification of mature eggs shows a strong increase in retention (*** significant via unpaired Student’s *t*-test, *P*=0.0005). Mature eggs were defined as stage 14 egg chambers with dorsal appendage and no NCs remaining. (D) Quantification of eggs laid show a significant decrease in fecundity (** significant via unpaired Student’s *t*-test, *P*=0.0037). Scale bar: 100 µm. Each data point represents a range of eight to ten females. Raw data in Table S1. Created in BioRender by Dixon, C. (2025). https://BioRender.com/p95q080. This figure was sublicensed under CC-BY 4.0 terms.

Previously, we demonstrated that *Drosophila* show increased egg retention following mild trauma (Dixon and McCall, 2023). Here we investigated whether increasing the severity of TI led to more severe reproductive consequences. To control the amount of TI, we used the high impact trauma device (Katzenberger et al., 2015), and subjected flies to five consecutive hits compared to one hit used previously to administer mild trauma. After subjecting flies to trauma, we examined two aspects of reproduction: ovarian health and fecundity. Females that underwent severe trauma displayed about a threefold increase in mature egg retention in comparison to sham counterparts (Fig. 1A-C). Additionally, the number of eggs laid per female (fecundity) was found to be significantly decreased in comparison to sham females (Fig. 1D), with trauma females laying approximately 23% less eggs compared to sham females. These effects on egg retention and fecundity were more severe than what was previously found in mild trauma females (Dixon and McCall, 2023). Additionally, we observed a heightened rate of death seen in females that underwent severe trauma (Fig. S1).

### Injured females displayed increased rates of midstage egg chamber death

**Fig. 2.**
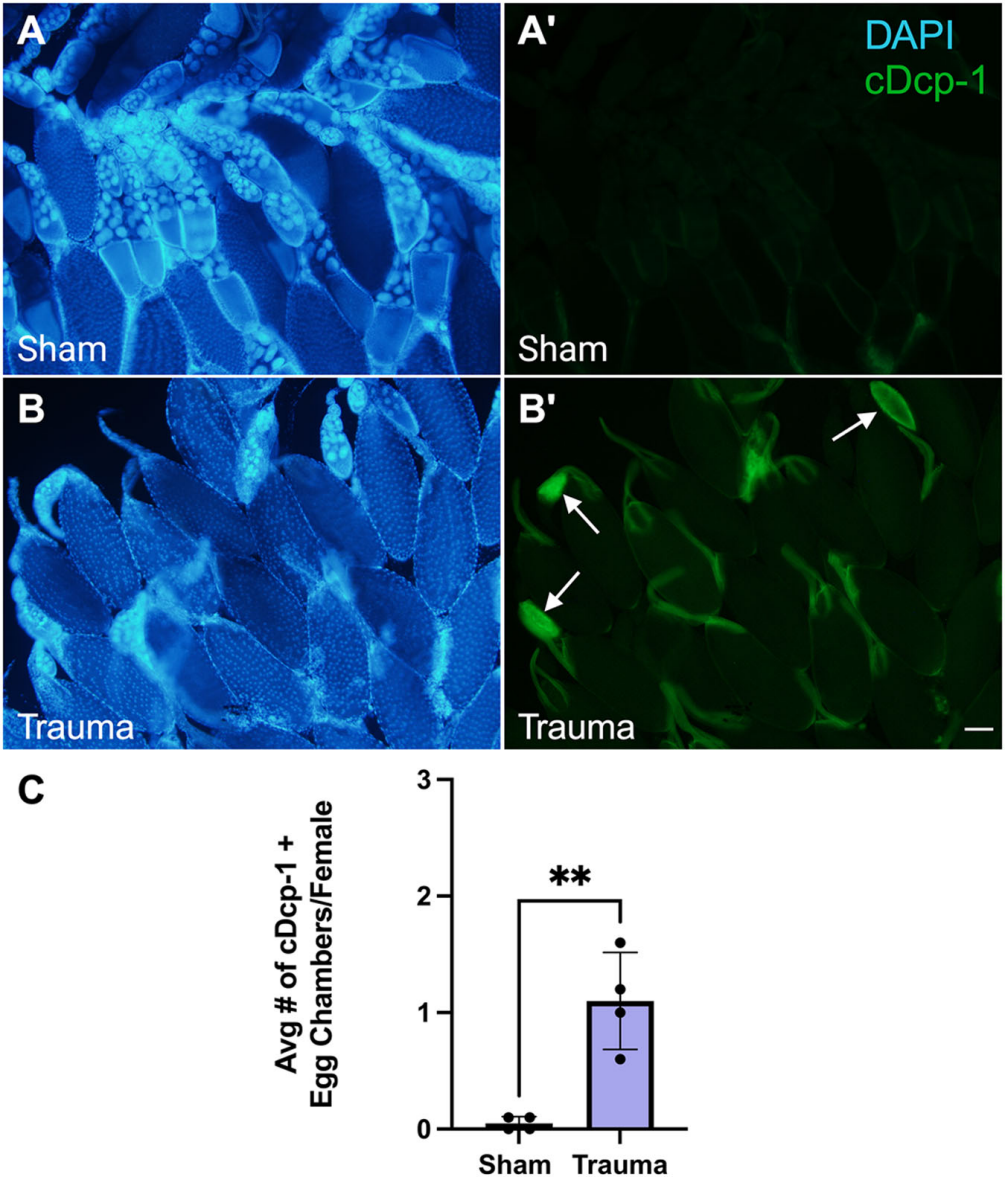
**Severe trauma leads to midstage death in the ovary.** (A) Ovaries from sham females (5 dpt) stained with DAPI (blue). (A′) cDcp-1 (green) labeling shows little caspase activity in sham females. (B) Ovaries from trauma females (5 dpt) stained with DAPI. (B′) cDcp-1 labeling of ovaries from trauma females. Examples of cDcp-1 positive egg chambers are marked with arrows. (C) Quantification of cDcp-1 positive midstage egg chambers shows a significant increase in trauma females versus sham counterparts (** significant via unpaired Student’s *t*-test, *P*=0.0025). Scale bar: 100 µm. Each data point represents an average of ten females. Raw data in Table S2. Created in BioRender by Dixon, C. (2025). https://BioRender.com/n75k050. This figure was sublicensed under CC-BY 4.0 terms.

While examining DAPI-stained ovaries for previously known phenotypes, a new phenotype of midstage egg chamber death was found. Mid-oogenesis plays an important transitional role for the overall development within the ovariole. Acting as a final checkpoint for developmental abnormalities, nutritional needs, and other environmental factors, midstage egg chambers can either progress onto later stages or they are eliminated by cell death (Lebo and McCall, 2021). To visualize apoptosis, we stained ovaries with anti-cleaved Dcp-1 (cDcp-1), which detects caspase activity (Sarkissian et al., 2014). Sham females displayed low amounts of midstage death (Fig. 2A-A′). Trauma females showed significantly higher amounts of cDcp-1 positive midstage egg chambers (Fig. 2B-B′,C). Affected egg chambers ranged from stage 7 to stage 9, corresponding to the onset of vitellogenesis and the mid-oogenesis checkpoint (Drummond-Barbosa and Spradling, 2001). These findings demonstrate that exposure to severe trauma leads to increased ovarian cell death similar to other environmental stresses.

### Trauma related midstage death is dependent upon Dcp-1

**Fig. 3.**
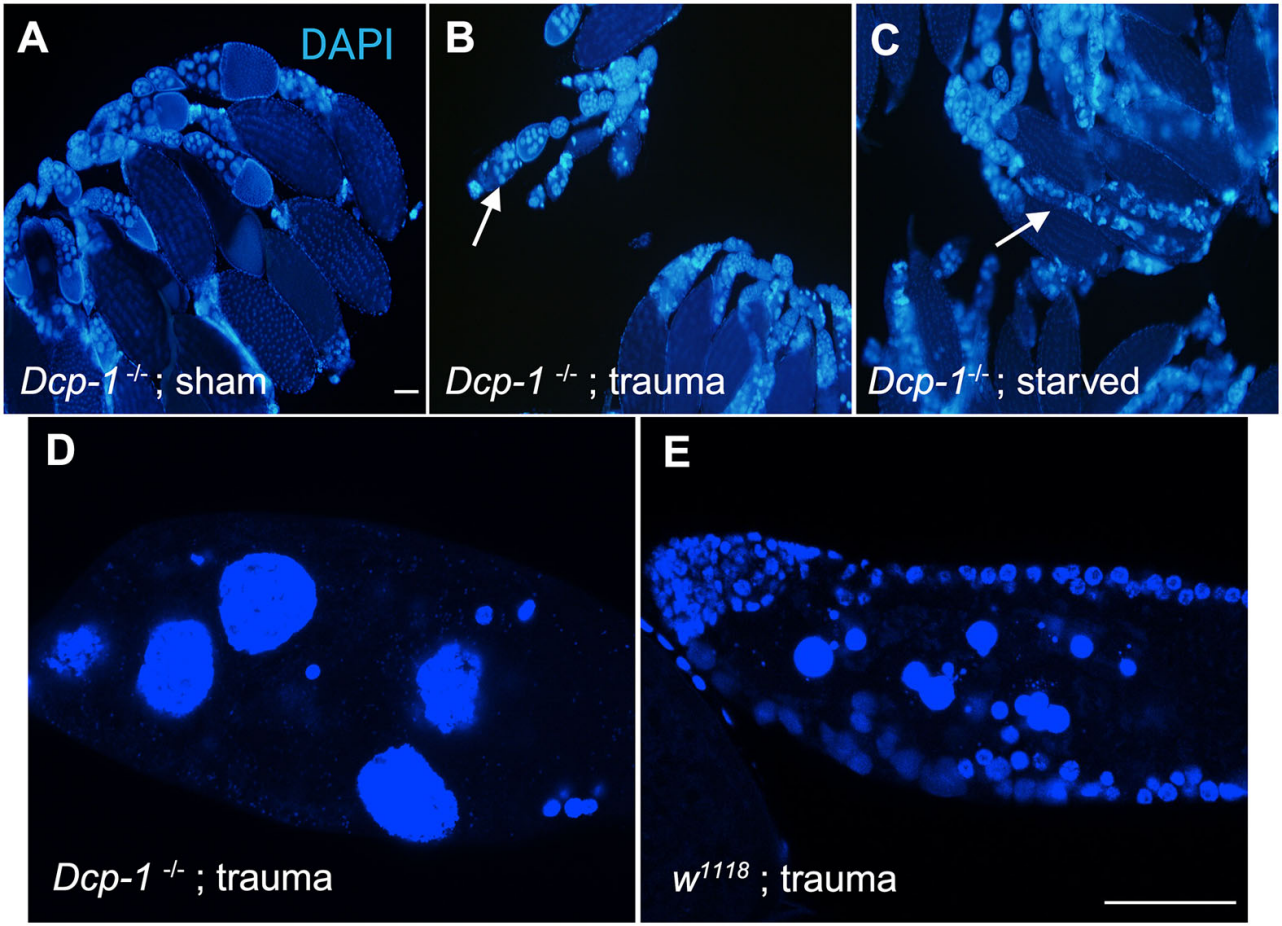
**Midstage death occurring from trauma is Dcp-1-dependent.** (A) Ovaries from sham *Dcp-1* null mutants stained with DAPI. (B) Ovaries from trauma *Dcp-1* null mutants (5 dpt). (C) Ovaries from starved *Dcp-1* null mutants. Arrows indicate representative regions with undead or PWOP egg chambers. (D) Confocal image a PWOP from *Dcp-1* null mutants that underwent trauma. (E) Confocal image depicting a degenerating egg chamber from *w^1118^* female that underwent trauma. (A-C) Scale bar: 100 µm. (D,E) Scale bar: 50 µm. Confocal images were brightened in FIJI for visualization. Created in BioRender by Dixon, C. (2025). https://BioRender.com/j99r096. This figure was sublicensed under CC-BY 4.0 terms.

Given the morphological similarity of midstage egg chamber death caused by trauma to that caused by protein starvation (Drummond-Barbosa and Spradling, 2001), we investigated whether egg chamber death that occurs from trauma utilizes the same apoptotic pathway. Starvation-induced cell death is dependent on the caspase Dcp-1 (Laundrie et al., 2003). Wild-type degenerating egg chambers display fragmentation and condensation of NC nuclei followed by phagocytosis by surrounding FCs. However, *Dcp-1* mutants exhibit an undead egg chamber phenotype, also known as peas without pods (PWOPs), where there is a failure of NC condensation and disappearance of the FCs (Baum et al., 2007). Thus, we exposed *Dcp-1* mutant females to trauma and compared egg chamber morphology to mutant sham counterparts. *Dcp-1* mutant females that underwent trauma did not display any midstage egg chamber degeneration (Fig. 3E), however, they had multiple undead egg chambers similar to starvation (Fig. 3B-D). These findings indicate that trauma-induced cell death in the ovary occurs via *Dcp-1*-dependent apoptosis, similar to the starvation pathway. Interestingly, *Dcp-1* mutants did not display significantly reduced fecundity or increased egg retention in response to trauma (Fig. S2). These findings suggest that midstage cell death is linked to other reproductive deficits.

### Trauma leads to higher rates of dying germ cell cysts in the germarium

**Fig. 4.**
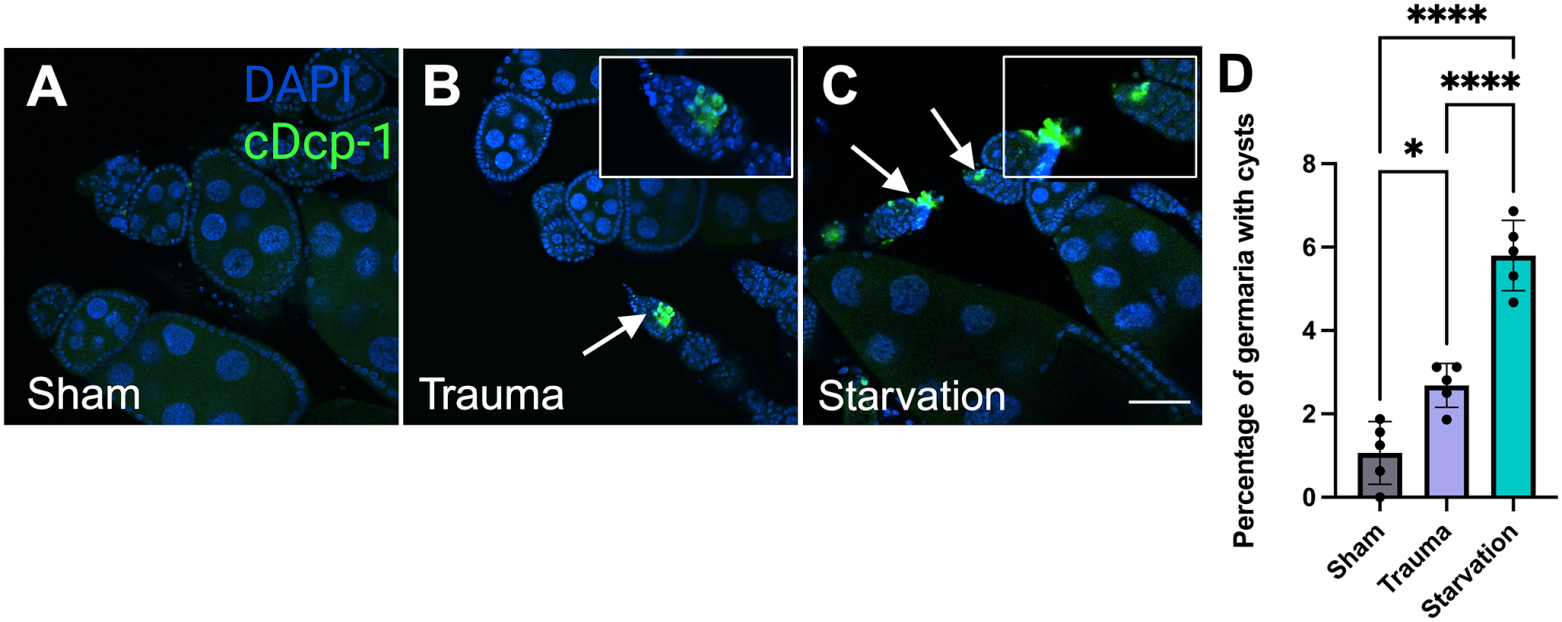
**Trauma induces cell death in the germarium similar to starvation.** (A) Confocal imaging of egg chambers from sham females (*w^1118^*) stained with DAPI and cDcp-1. (B) Confocal image of egg chambers from trauma *w^1118^* females stained with DAPI and cDcp-1 with an arrow marking an example of cell death in the germarium. (C) Confocal image of egg chambers from starved *w^1118^* females stained with DAPI and cDcp-1 with arrows marking examples of cell death in the germarium. (D) Quantification of dying cysts found in germaria. There were significantly more dying cysts in the starvation group when compared to sham and trauma. Trauma had significantly more dying cysts than sham but not as many as starvation controls (**** significant via one-way ANOVA, *P*=0.0001; multiple comparisons with Tukey's multiple comparison test: *=0.0101 *P*-value, ****=0.0001 *P*-value). Scale bar: 50 µm. Zoomed in images of where arrow is pointing shown in white boxes. Confocal images were brightened (DAPI only) in FIJI for visualization. Each data point represents an average of ten females. Raw data in Table S3. Created in BioRender by Dixon, C. (2025). https://BioRender.com/r14x343. This figure was sublicensed under CC-BY 4.0 terms.

Upon starvation conditions, cell death also occurs in early oogenesis, primarily of developing germ cell cysts in the germarium (Drummond-Barbosa and Spradling, 2001). To determine if cell death in early oogenesis also occurs in response to trauma, we examined ovaries stained with anti-cDcp-1. Higher rates of cDcp-1 staining were observed in the germaria and early-stage egg chambers of trauma females versus their sham counterparts (Fig. 4). To determine how the observed ovary phenotypes compared to starvation, we scored the presence of cell death between sham, trauma, and starvation controls, which canonically have high rates of death in the germarium. While trauma groups had significantly higher rates of death in the germarium compared to sham, they exhibited lower rates than starved controls (Fig. 4D).

### Female reproductive defects have an inverse relationship with each other over time

**Fig. 5.**
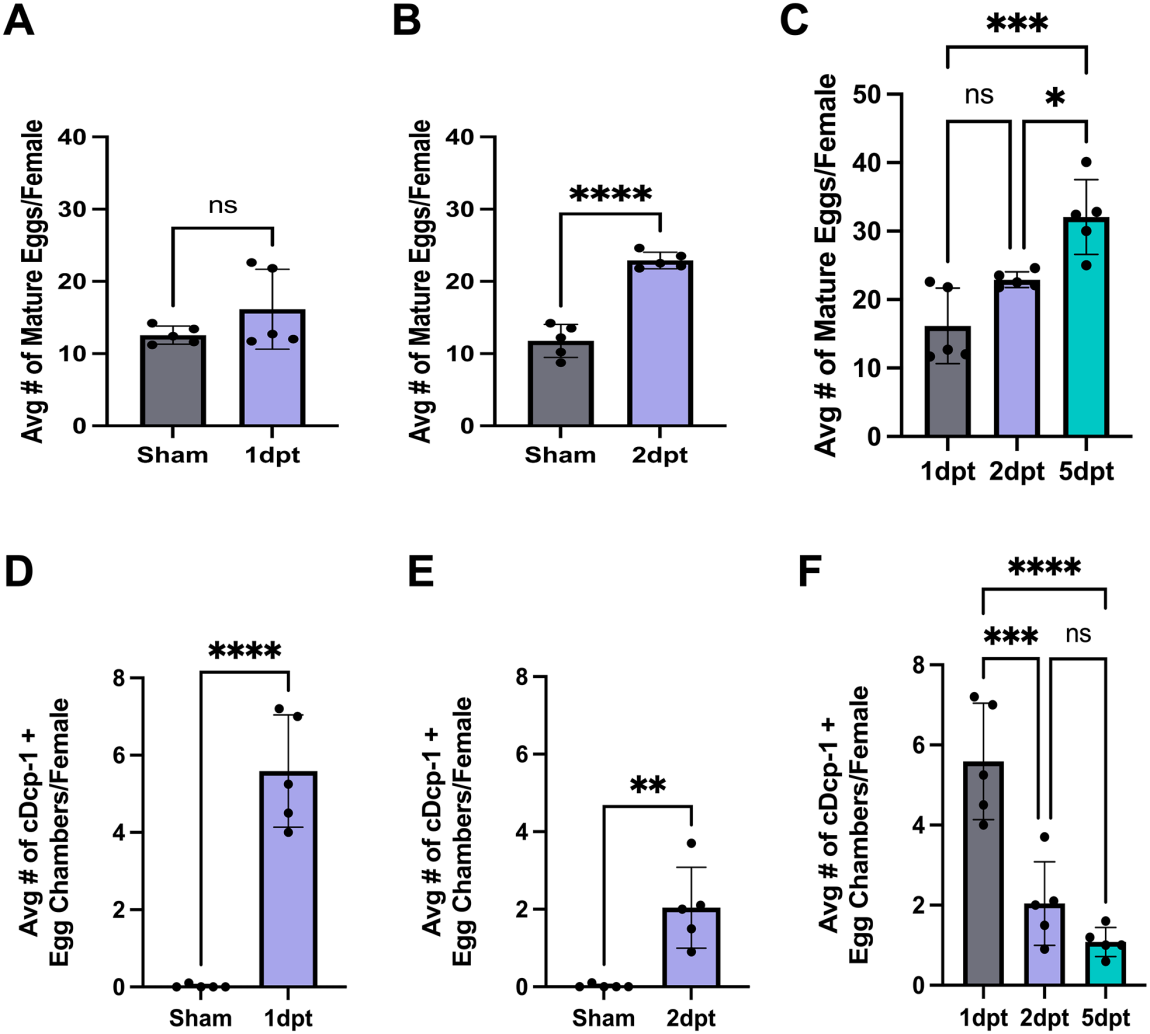
**Timecourse of reproductive phenotypes shows inverse relationship between egg retention and cell death.** (A) Quantification of the number of mature eggs retained per female (1 dpt) was not significantly different than sham (via unpaired Student’s *t*-test, *P*=0.1935). (B) The number of eggs retained per female (2 dpt) was significantly higher when compared to sham (**** significant via unpaired Student’s *t*-test, *P*=0.0001). (C) Comparison between 1, 2, and 5 dpt shows that retention significantly increases over time with 5 dpt showing the highest number of retained eggs (*** significant via ordinary one-way ANOVA, *P*=0.0005; Tukey's multiple comparison test: ns=0.0869 *P*-value, *=0.0197, ***=0.0003). (D) Quantification of the amount of midstage death per female (1 dpt) shows significant increase between sham and trauma (**** significant via unpaired Student’s *t*-test, *P*=0.0001). (E) The amount of midstage death per female (2 dpt) was also higher (** significant via unpaired Student’s *t*-test, *P*=0.0025). (F) Comparison across days shows midstage death is significantly highest at 1 dpt and then lessens overtime. (**** significant via ordinary one-way ANOVA, *P*=0.0001; Tukey's multiple comparison test: ns=0.3516 *P*-value, ***=0.0005, ****=<0.0001). Each data point represents an average of ten females. Raw data in Table S4. Created in BioRender by Dixon, C. (2025). https://BioRender.com/h61r312. This figure was sublicensed under CC-BY 4.0 terms.

Our initial analysis was conducted 5 days post trauma (dpt) when both heightened rates of retention of mature eggs and midstage death were observed. To tease apart the timing of onset for reproductive phenotypes, a timecourse was performed. Females were dissected on various days (1, 2, and 5 dpt) and phenotypes were compared. Retention of mature eggs reached a significant increase by day two and continued to increase through 5 days (Fig. 5A-C). Inversely, midstage death appeared to be at its highest rate early on day one and decreased by day five (Fig. 5D-F). These findings indicate that the observed midstage death is not dependent on egg retention and is an acute response to trauma.

### Injured females develop melanization, which is not correlated with the severity of reproductive defects

**Fig. 6.**
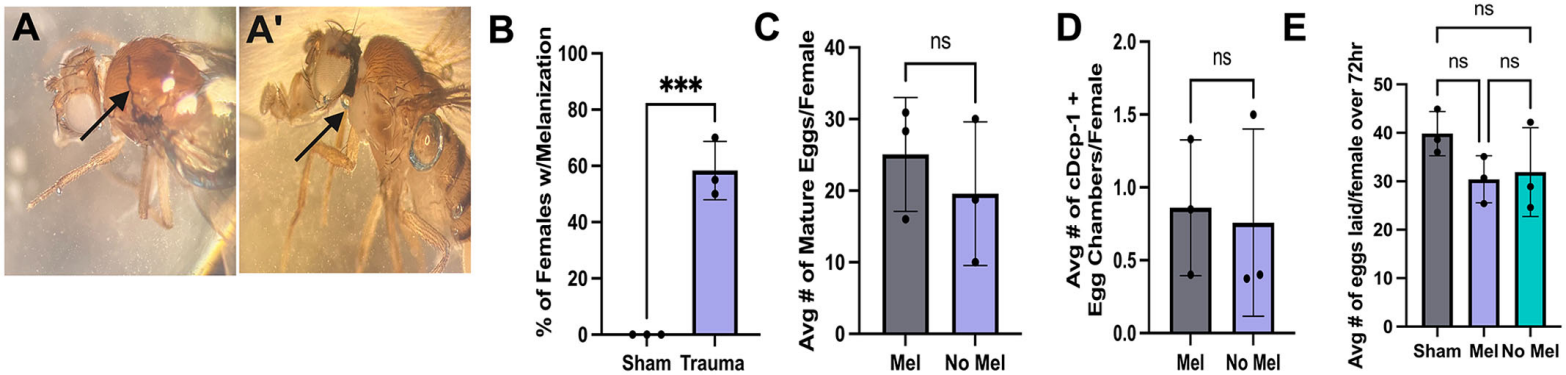
**Severe trauma leads to development of melanization.** (A-A′) Images show melanization present on females 5 dpt (noted by arrow). (B) Quantification of the percentage of females that develop melanization post-trauma showed a strong difference between sham and trauma (*** significant via unpaired Student’s *t*-test, *P*=0.0006). (C) Quantification of the average number of mature eggs per female was not significantly different between injured females with and without melanization (not significant via unpaired Student’s *t*-test, *P*=0.4993). (D) Quantification of the average amount of midstage death per female was not significantly different between injured females with and without melanization (not significant via unpaired Student’s *t*-test, *P*=0.8352). (E) Quantification of the average number eggs laid per female for a 72 h period (not significant via one-way ANOVA, *P*=0.2447, Šidák's multiple comparisons, *P*=0.3375, *P*=0.4656, *P*=0.9905). Each data point represents an average of ten females. Raw data in Table S5. Created in BioRender by Dixon, C. (2025). https://BioRender.com/g01q290. This figure was sublicensed under CC-BY 4.0 terms.

Over the course of this study, females were noted to develop melanization at a significantly higher rate than their sham counterparts (Fig. 6A-C). Previously Lika et al. reported that larvae that had undergone trauma developed melanization, which correlated with shortened lifespan as adults (Lika et al., 2021). To determine if melanization in adult females could be used as a noninvasive indicator of reproductive deficits, both females (with and without melanization) post-trauma were compared. These groups were not significantly different from each other in either retention or midstage death (Fig. 6C,D), suggesting that the reproductive deficits were not due to external injuries. Moreover, the average fecundity rate was not significantly different between flies with and without melanization (Fig. 6E).

## DISCUSSION

TIs lead to many homeostatic disruptions that result in a variety of pathologies that can develop many years post-injury. A lesser-known consequence of TI, specific to females, are reproductive deficits related to egg production and maintenance; however, the causes of this are not well understood (Ripley et al., 2008). While *Drosophila* have been shown to be an excellent model to study pathologies post-trauma (Buhlman et al., 2021; Katzenberger et al., 2015), female-specific reproductive consequences after injury had not been investigated until recently. We previously showed that mild global trauma leads to ovulation defects, in the form of mature egg retention (Dixon and McCall, 2023). In the current study, we aimed to investigate the defects that result from increased trauma.

Females that were subjected to trauma were compared to sham counterparts for the same assays that were performed in our previous study: ovarian analysis for retention phenotypes and fecundity. Post-injuries, females were observed to have higher rates of retention than sham counterparts (Fig. 1) and mild trauma (Dixon and McCall, 2023). Additionally, trauma females showed decreased egg laying compared to controls and exhibited significantly higher rates of retention compared to sham counterparts (Fig. 1), whereas under mild trauma we found a modest decrease in egg laying that did not reach significance (Dixon and McCall, 2023).

Interestingly, while examining ovaries for retention phenotypes we observed higher rates of midstage egg chamber death compared to sham, a phenotype that was not seen with mild trauma. This was confirmed using immunohistochemistry against cleaved (activated) caspase Dcp-1 (Meehan et al., 2015; Sarkissian et al., 2014) and cDcp-1+ egg chambers were found to occur at a significantly higher frequency in trauma females compared to their sham counterparts (Fig. 2).

Given the two phenotypes of increased egg retention and cell death, the next question was the classic chicken and egg, whether retention was a result of midstage death or whether retention of mature eggs led to increased cell death in younger stages of egg chambers. Alternatively, they could occur independently of each other. To answer this, a timecourse was utilized that examined three time points over a 5-day period. Retention was found to increase over the timecourse, but midstage death was high early post-trauma and then lessened over time (Fig. 4). This suggests that trauma leads to early midstage death, which could lead to the retention of mature eggs and a decrease in fecundity. This is supported by our findings that *Dcp-1* mutants, which display defective midstage death, did not show effects on fecundity or egg retention in response to trauma. However, previously we found that wild-type flies following mild trauma showed egg retention without an increase in cell death suggesting the two phenotypes can be independent (Dixon and McCall, 2023). Indeed, a recent report has found distinct signaling pathways acting in the fat body to promote these phenotypes (Bradshaw et al., 2024). Determining whether the fat body mediates any response to trauma is a particularly intriguing avenue for future study. It takes approximately 10 days for egg chambers to progress from stem cells to mature eggs (Horne-Badovinac, 2014; Spradling, 1993), which suggests that the retained eggs are both pre-injury and post-injury. Additionally, nutrient deprivation causes delays in egg chamber growth and progression and there may be similar influences from trauma (Shimada et al., 2011).

The phenotypes that were exhibited post-trauma are reminiscent of starvation related phenotypes (Drummond-Barbosa and Spradling, 2001; Lebo and McCall, 2021; Pritchett and McCall, 2012). To determine whether trauma acted through the same apoptotic pathway in mid-oogenesis, we examined a *Dcp-1* null mutant and found that post-trauma, these mutants did not exhibit degenerating egg chambers, confirming that trauma induced cell death was dependent on *Dcp-1*, similar to starvation (Fig. 3). The second approach to determine if the phenotypes from trauma were similar to starvation was to examine the rate of dying germline cysts found in the germarium. The presence of these clusters is higher in ovaries of starved flies (Armstrong and Drummond-Barbosa, 2018; Drummond-Barbosa and Spradling, 2001). Through confocal microscopy, the number of clusters found in the germaria, and early oogenesis was found to be significantly higher in trauma groups compared to their sham counterparts (Fig. 4). However, the frequency of these clusters was substantially less than what was previously reported for starvation (Armstrong and Drummond-Barbosa, 2018; Drummond-Barbosa and Spradling, 2001). Observing cell death in both midstage egg chambers and within the germaria in response to trauma gives insight into these key checkpoints in ovary development. Flies were observed to be eating and producing waste, which suggests that starvation conditions were not occurring in traumatized flies (Katzenberger et al., 2015). The Dcp-1 pathway acts as a common response to many environmental stresses (Kim et al., 2010) and while the reproductive phenotypes displayed may be similar, the activation and utilization of the pathway may be different.

Some females that underwent severe trauma developed cuticle melanization as a result of the injuries. Melanization, in arthropods, is a result of crystal cell activation to envelop and remove pathogens from the organism or respond to cuticular injury (Tang, 2009; Vlisidou and Wood, 2015). Following trauma, melanization has been shown to occur in larval *Drosophila* and was correlated with shortened lifespan (Lika et al., 2021). We hypothesized that melanization development could indicate severity of trauma and be linked to reproductive phenotypes, thus providing a non-invasive marker of female reproductive detriments. However, females that developed melanization post-trauma did not display any more severe ovary phenotypes than injured females that did not develop melanization (Fig. 6). While melanization may be a marker of injury or immune response, it does not appear to be a reliable predictor for whether the injured females will have reproductive phenotypes.

Understanding the impact of TIs on female reproduction is vital to fleshing out the physiological response to trauma. While vertebrate models have provided excellent sources of information on responses to trauma, increasing the scope of models to include invertebrate counterparts are invaluable. While there are different aspects of reproduction between humans and flies, *Drosophila*, and other invertebrates, offer a low cost, fast, and ethically more appealing model to understand these pathologies. Overall, studies and models of female reproductive consequences post trauma are lacking, despite the documented effects in humans (Ripley et al., 2008). We have established *Drosophila* as a model for the investigation of female reproductive detriments post-trauma and have demonstrated that several phenotypes arise. However, the mechanism(s) that lead to reproductive deficits remain to be determined. Key tissues to investigate are immune and hormonal signaling via the brain, gut and/or fat body, and the wealth of tools in *Drosophila* will allow for the dissection of these mechanisms. In mammals, it has been observed that brain perturbations can lead to the reproductive defects seen in females due to disruption of major hormonal axes, which warrants comparisons in the invertebrate model (Joseph and Whirledge, 2017). In addition to testing the hormone hypothesis, a proper examination into connections with starvation/nutrition are crucial to fleshing out the overall issues in multisystem disease. Moreover, longer term studies are needed to determine if reproductive deficits persist over the lifetime of the fly. *Drosophila* are an invertebrate model that can be leveraged to investigate female reproductive consequences post-trauma and provide a basic biology approach to shed light on how TIs effect humans.

## MATERIALS AND METHODS

### Fly strains

Flies were raised on a cornmeal-yeast diet at 25°C with 12-h day-light cycle. Predominantly, the control strain *w^1118^* was used for experiments (retention, midstage death, and germarium assays). For assays used to confirm involvement of the Dcp-1 pathway, the *Dcp-1^prev1^* null mutant (Laundrie et al., 2003) was utilized.

### High impact trauma (HIT)

The HIT device was constructed in the Biology Workshop at Boston University using the parameters described (Katzenberger et al., 2015). Ten flies were placed in an empty vial with a 1-inch allotment of space. A plug was placed at the 1-inch line from the bottom of the vial and loaded onto the HIT device (Katzenberger et al., 2015). All experiments were performed at 90° with five strikes of the device per group and given supplemental yeast paste 2 days prior to injury.

### Egg laying assay

Mated females were raised to 5 days post eclosion (dpe) along with seven to ten males with supplemental yeast paste given daily beginning on 3 dpe. Groups of ten females were then either subjected to trauma via HIT device or were left unharmed (sham). Females were paired with the same males from the raising period and placed in the egg laying apparatus, which consisted of an overturned bottle sealed with a plate containing grape juice agar and a dollop of yeast paste. The grape agar color made for easy visualization of eggs while the supplemental yeast paste was to reduce nutrition effects. Egg laying was conducted over a 4-day period with counts performed over the center 72-h period to allow time for acclimation. Each day during the timecourse, plates were collected and counted for eggs laid. This assay consisted of three replicates, each consisting of ten females, for a total of 30 females observed for both conditions.

### Immunohistochemistry

Dissection of ovaries was performed using forceps to extract whole ovaries from females (ten females). Samples were fixed immediately post-dissection in 4% paraformaldehyde (PFA). Fixed samples were then washed in 1x phosphate buffered saline with 0.1% Triton-X (1xPBT). For DAPI staining, washed samples were covered in Vectashield with DAPI (Vector Labs) and incubated at 4°C overnight prior to mounting on slides. For cDcp-1 staining, washed samples were blocked in 1xPBT containing 0.5% bovine serum albumin (BSA) for 1 h at room temperature. Primary antibody incubation was performed using PBT/BSA with 5% normal goat serum (PBANG). The primary antibody used was rabbit α-cleaved Dcp-1 (Cell Signaling Technology #9578, 1:100 concentration) and samples were incubated overnight at 4°C. Primary was removed and samples were washed several times with 1xPBT/0.5%BSA. Secondary antibody used was goat α-rabbit alexa-fluor 488 (Jackson ImmunoResearch 111-545-003, 1:1000 concentration) and samples were incubated for 1 h at room temperature. Secondary was removed and samples were washed in 1xPBT/.5%BSA and then incubated in Vectashield with DAPI overnight at 4°C. Samples were then placed and sealed onto slides.

### Microscopy

Experiments were conducted using one of three different microscopes. The majority of imaging data was collected via an epifluorescence microscope (Olympus BX60), including retention and midstage death assays. Images of melanization on females was conducted via dissecting microscope. Imaging of germaria was performed via confocal microscopy (Nikon Ti2). Quantifications were made by scanning/scoring the slides individually. Confocal images were brightened (only DAPI) using the adjust brightness function in FIJI.

### Analysis

Statistical analyses were performed using GraphPad Prism (V10.2.3) (unpaired Student’s *t*-tests and one-way ANOVA). Multiple comparisons made in addition with one-way ANOVA was done via Tukey's multiple comparisons test. Confocal images were processed in FIJI and the figures were created using BioRender.com.

## Supplementary Material

10.1242/biolopen.061825_sup1 Supplementary information
