# Supplementary material for: Traumatic injury leads to ovarian cell death and reproductive disturbances in Drosophila melanogaster
Source: Biol Open. 2025 Feb 24;14(2):BIO061825. doi: 10.1242/bio.061825 (PMC11892359; doi:10.1242/bio.061825)
Supplement: Supplementary information [file biolopen-14-061825-s1.pdf]

Fig. S1

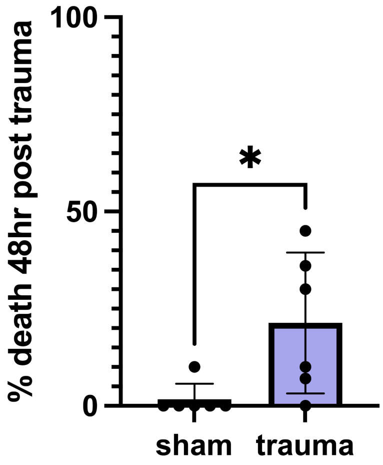

Fig. S1. Severe trauma leads to short term heightened mortality Quantification of post-severe trauma mortality in *w*^{1118} control females (significant via unpaired t test, p=.0267). Created in BioRender by Dixon, C. (2025). https://BioRender.com/i38j377. This figure was sublicensed under CC-BY 4.0 terms.

Fig. S2

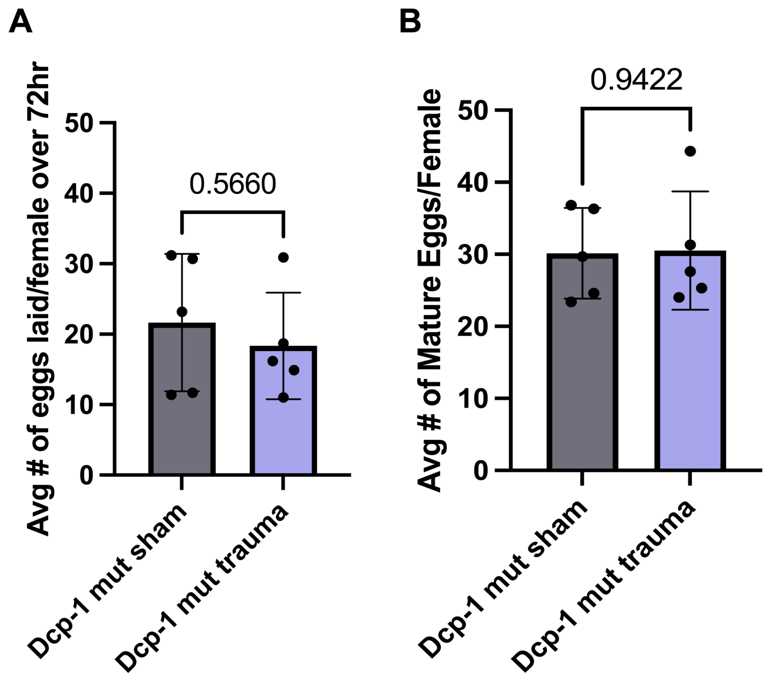

Fig. S2. Reproductive phenotypes are not enhanced post-trauma in *Dcp-1* mutants (A) Quantification of eggs laid over 72 hours from *Dcp-1* mutant females post-severe trauma (non-significant via unpaired t test, p=.5660). (B) Quantification of mature eggs retained from *Dcp-1* mutant females post-severe trauma (non-significant via unpaired t test, p=.9422). Created in BioRender by Dixon, C. (2025). https://BioRender.com/d01f146. This figure was sublicensed under CC-BY 4.0 terms.

Table S1. Raw data corresponding to Fig. 1

Panel C:

Egg retention

sham # of st14s	# females	sham # st14s/female	trauma # of st14s	# females	trauma # st14s/female
124	10	12.4	234	10	23.4
132	10	13.2	250	10	25
150	10	15	370	10	37
130	10	13	310	10	31
124	10	12.4	175	10	17.5
116	10	11.6	328	10	32.8

Panel D:

Egg laying

sham # of eggs Day2/female	sham # of eggs Day3/female	sham # of eggs Day 4/female	sham mean # eggs/female	trauma # of eggs Day2/female	trauma # of eggs Day3/female	trauma # of eggs Day4/female	trauma mean # eggs/female
41.4	40	40.7	40.7	33.5	29.1	30	30.8
45	48	42	45	31	30	29.8	30.3
46	45.3	43.7	45	25	26	22.5	24.5

Table S2. Raw data corresponding to Fig. 2

Panel B:

Midstage death

sham # cDcp-1+ egg chambers	# females	sham # cDcp-1+/female	trauma # cDcp-1+ egg chambers	# females	trauma # cDcp-1+/female
1	10	0.1	6	10	0.6
0	10	0	10	10	1
0	10	0	12	10	1.2
1	10	0.1	16	10	1.6

Table S3. Raw data corresponding to Fig. 4

Panel D:

Dying cysts in germaria

sham # cDcp-1+ cysts	Avg # of ovarioles	sham mean % cDcp-1+ cysts	trauma # cDcp-1+ cysts	Avg # of ovarioles	trauma mean % cDcp-1+ cysts	starvation # cDcp-1+ cysts	Avg # of ovarioles	starvation mean % cDcp-1+ cysts
1	160	0.63	5	160	3.125	8	160	5.31
0	160	0	4	160	2.5	7	160	4.68
2	160	1.25	5	160	3.125	9	160	5.9
3	160	1.875	3	160	1.86	10	160	6.25
3	160	1.56	4	160	2.81	11	160	6.86

Biology Open • Supplementary information

Table S4. Raw data corresponding to Fig. 5

Panel A:

Sham # st14s	Sham # st14s/ female	1dpt* # of st14s	1dpt # st14s/ female
124	12.4	127	12.7
116	11.6	226	22.6
134	13.4	218	21.8
142	14.2	117	11.7
112	11.2	120	12

Panel B:

Sham # st14s	Sham # st14s/ female	2dpt # st14s	2dpt # st14s/ female
142	14.2	221	22.1
87	8.7	235	23.5
122	12.2	218	21.8
135	13.5	225	22.5
102	10.2	246	24.6

Panel C:

1dpt # st14s	1dpt # st14s/female	2dpt # st14s	2dpt # st14s/female	5dpt # st14s	5dpt # st14s/female
127	12.7	221	22.1	324	32.4
226	22.6	235	23.5	300	30
218	21.8	218	21.8	401	40.1
117	11.7	225	22.5	250	25
120	12	246	24.6	328	32.8

Panel D:

Sham # cDcp-1+ egg chambers	Sham # cDcp-1+/female	1dpt # cDcp-1+ egg chambers	1dpt # cDcp-1+/female
0	0	72	7.2
1	0.1	40	4
0	0	70	7
0	0	53	5.3
0	0	45	4.5

Panel E:

Sham # Dcp-1+ egg chambers	Sham # cDcp-1+/female	2dpt # Dcp-1+ egg chambers	2dpt # cDcp-1+/female
0	0	37	3.7
1	0.1	9	0.9
0	0	21	2.1
0	0	15	1.5
0	0	20	2

Panel F:

1dpt # Dcp-1+ egg chambers	1dpt # cDcp-1+/female	2dpt # Dcp-1+ egg chambers	2dpt # cDcp-1+/female	5dpt # Dcp-1+ egg chambers	5dpt # cDcp-1+/female
72	7.2	37	3.7	6	0.6
40	4	9	0.9	10	1
70	7	21	2.1	12	1.2
53	5.3	15	1.5	16	1.6
45	4.5	20	2	10	1

* dpt: days post trauma

Table S5. Raw data corresponding to Fig. 6

Panel B:
Melanization

Sham # flies with mel	Sham % flies with mel	Trauma # of flies with mel	Trauma % flies with mel
0	0	5	50
0	0	7	70
0	0	5.5	55

Panel C:
Egg retention

Mel flies # of st14s	Mel # st14s/female	No Mel flies # of st14s	No Mel # st14s/female
283	28.3	100	10
160	16	300	30
309	30.9	187	18.7

Panel D:
Midstage death

Mel # cDcp-1+ egg chambers	# Mel females	Mel # cDcp-1+ /female	No Mel # cDcp-1+ egg chambers	# No Mel females	No Mel # cDcp-1+ /female
16	12	1.33	4	10	0.4
4	10	0.4	18	12	1.5
6	7	0.9	3	8	0.375

Panel E:
Egg laying

Sham # of eggs Day2/female	Sham # of eggs Day3/female	Sham # of eggs Day4/female	Sham mean # eggs/female	Mel # of eggs Day2/female	Mel # of eggs Day3/female	Mel # of eggs Day4/female	Mel mean # eggs/female	No Mel # of eggs Day2/female	No Mel # of eggs Day3/female	No Mel # of eggs Day4/female	No Mel mean # eggs/female
36.3	39.8	32	36	30.2	25.8	20.2	25.4	25.9	31.4	16.4	24.6
45.8	40.4	48.7	44.9	31.3	30.1	30.8	30.7	36	23.7	27	28.9
35.7	43.3	36.9	38.6	35.6	32.9	36.7	35.1	39.1	45.6	41.8	42.2

Mel= flies with melanization
No Mel = flies without melanization

Biology Open • Supplementary information

Table S6. Raw data corresponding to Fig. S1

sham<br># of deaths	# females	sham<br>% death	trauma<br># of deaths	# females	trauma<br>% death
0	10	0	1	10	10
1	10	10	0	10	0
0	10	0	3	10	30
0	10	0	5	14	36
0	10	0	5	11	45
0	10	0	1	15	7

Table S7. Raw data corresponding to Fig. S2

Panel A:
Egg laying

Dcp-1 sham Day2<br># eggs/female	Dcp-1 sham Day3<br># of eggs/female	Dcp-1 sham Day4<br># of eggs/female	Dcp-1 sham<br>mean #	Dcp-1 trauma Day2<br># eggs/female	Dcp-1 trauma Day 3<br># eggs/female	Dcp-1 trauma Day4<br># eggs/female	Dcp-1 trauma<br>mean #
10.9	11.1	12.3	11.4	31.7	6.5	10.5	16.2
22.4	35.5	35.8	31.2	24.1	13.3	18.7	18.7
16.1	10.7	8.3	11.7	8.4	10.2	14.5	11
27.7	24.2	17.6	23.2	34.2	32.5	25.9	30.9
46.3	29.8	15.9	30.7	20.1	13.7	11	14.9

Panel B:
Egg retention

Dcp-1 sham<br># st14s	# females	Dcp-1 sham<br># st14s/female	Dcp-1 trauma<br># st14s	# females	Dcp-1<br># st14s/female
221	6	36.8	243	10	24.3
197	8	24.6	219	7	31.3
363	10	36.3	276	10	27.6
187	8	23.4	202	8	25.3
297	10	29.7	399	9	44.33

Biology Open • Supplementary information
